# Phase Angle in Bioelectrical Impedance: New Perspectives in Health and Body Composition Assessment

**DOI:** 10.33549/physiolres.935629

**Published:** 2025-12-01

**Authors:** Jitka JIRKŮ, Jarmila KŘÍŽOVÁ

**Affiliations:** 1 3rd Department of Internal Medicine, Department of endocrinology and metabolism, General University Hospital in Prague, 1st Faculty of Medicine, Charles University, Prague, Czech Republic; 2 Nutrition Outpatient Clinic, Prague, Czech Republic

**Keywords:** Bioelectrical impedance analysis, Phase angle, Body composition, Sex characteristics, Body water distribution

## Abstract

The aim of this observational, descriptive, and cross-sectional study was to analyze the relationship between phase angle (PhA), determined by bioelectrical impedance analysis (BIA), and body composition parameters in healthy adult individuals. The study included 265 participants (122 women and 143 men) aged 18–77 years, examined at a nutritional outpatient clinic in Prague between April 2022 and December 2023. Segmental multi-frequency BIA was performed using the Tanita MC-780 MA analyzer with eight electrodes and frequencies of 5 kHz, 50 kHz, and 250 kHz. The mean PhA values were significantly higher in men than in women (6.29° vs. 5.57°; p < 0.001). A strong negative correlation between the extracellular water-to-total body water ratio (ECW/TBW) and PhA was observed in men (r = −0.78; p < 0.001), whereas in women, the correlation was moderate (r = −0.33; p < 0.001). Conversely, a strong positive correlation was observed between PhA and intracellular water (ICW) volume in women (r = 0.71; p < 0.001), while in men this association was weaker (r = 0.17; p < 0.05). The data indicate that PhA is significantly correlated with body fluid distribution and body composition. The key determinants differ by sex–fat mass, particularly visceral fat, plays a predominant role in men, while in women, muscle mass appears to be the dominant factor. PhA thus emerges as a valid, non-invasive marker of body composition, sensitive to changes in internal milieu, with potential clinical applications for assessing nutritional status and the patient’s physiological condition.

## Introduction

Body composition analysis currently represents an integral part of both clinical and preventive medicine, enabling detailed quantification of individual body components and providing comprehensive information on a patient’s nutritional and metabolic status. Among the available methods, bioelectrical impedance analysis (BIA) is most commonly used in routine clinical practice due to its non-invasiveness, ease of use, rapidity, and relative accessibility. BIA is based on a two-compartment model of body composition, distinguishing fat mass (FM) and fat–free mass (FFM). FFM includes muscle tissue, organs, bone mass, and body water, which is further divided into intracellular (ICW) and extracellular (ECW) compartments [1]. A physiologically ideal ECW:ICW ratio is approximately 0.7–0.75; deviations from this ratio are observed in cases of edema, chronic inflammation, organ failure, or excess visceral fat [2].

The principle of BIA is based on the impedance of alternating electrical current - a complex variable comprising resistance (R) and reactance (Xc). Resistance reflects the flow of current through water with electrolytes and the ability of tissues to impede or stop the current. Adipose tissue has very high resistance, effectively blocking most of the current at the membranes of fat cells. Conversely, tissues with high water content, such as extracellular fluid and muscle tissue, exhibit low resistance. Reactance is related to the capacitive properties of cell membranes and represents the ability to slow down the current, which causes a phase shift [3].

One of the key BIA-derived parameters is phase angle (PhA), measured at the standard frequency of 50 kHz. PhA (expressed in degrees) is calculated using the formula: arctangent (Xc / R) × 180°/π, and typically ranges between 0 and 90 degrees. A PhA of 0° indicates no current flow through cell membranes, while 90° would indicate current passing without fluid resistance. PhA is considered a marker of cell membrane integrity, active body mass, and fluid distribution. In healthy individuals, higher PhA values are associated with greater physical fitness and muscle mass, increasing with improved conditioning and decreasing with muscle loss [4]. Normal PhA values range from 6° to 9°, depending on age, sex, height, and current health status. Higher PhA values are associated with intact cell membranes, higher ICW volume, and increased metabolic activity, whereas lower PhA values may indicate cellular damage, inflammation, malnutrition, or catabolic states [5,6].

Numerous studies have investigated PhA in various clinical conditions, such as sarcopenia, liver cirrhosis, dialysis, and critical illness, consistently demonstrating a decrease in PhA with increasing disease severity. Margain *et al*. [7] reported PhA cut-off values of 5.4° in women and 5.6° in men for diagnosing sarcopenia in patients with liver cirrhosis. In a study by Tsuji *et al*. [8], patients with chronic muscle pain and sarcopenia had significantly lower PhA values (3.9°) compared to non-sarcopenic individuals (4.9°). PhA is a reliable predictor of sarcopenia in cancer patients (e.g., stomach, colorectal, or prostate), but these findings may not generalize to non-oncological populations.

PhA is frequently associated with mortality and nutritional status, appears to be modulated by changes in nutritional state, declining in conditions such as cancer cachexia or malnutrition, and improving with appropriate nutritional interventions [1,9,10]; in support of this, a cohort of healthy obese individuals demonstrated an increase in PhA from 5.54° to 5.94° following reductions in body weight and fat mass [2]. PhA is increasingly being used not only as a diagnostic and monitoring tool for nutritional status but also as a prognostic marker in oncology, geriatrics, and chronic disease. Low PhA values have been associated with increased morbidity, longer hospital stays, and higher mortality [5,11].

In contrast to most existing studies, our research focused solely on a healthy adult population. The aim was to analyze the relationship between PhA and individual BIA parameters with regard to sex, and to identify the body composition components that most significantly influence PhA variability.

## Patients and Methods

This was an observational, descriptive, cross-sectional study conducted between April 2022 and December 2023. A total of 265 healthy individuals (122 women - 46 %, and 143 men - 54 %) aged 18 to 77 years who attended a clinical nutrition outpatient center in Prague were included. The study was approved by the Faculty of Medicine Ethics Board (Approval No. 224/21 S-IV) and all participants provided written informed consent prior to inclusion. Exclusion criteria included the presence of a cardiac pacemaker, age under 18 years, any acute or chronic illness, pregnancy, and lactation. Prior to their scheduled bioimpedance analysis (BIA) appointment, participants received instructions to refrain from food intake for at least 4 hours, avoid physical exercise or strenuous activity, and abstain from alcohol consumption on the day of measurement. Body height was measured using a calibrated stadiometer (Seca 206) with an accuracy of 0.5 cm. BIA was performed with participants standing upright, barefoot, and in light underwear, using a segmental multi-frequency body composition analyzer (Tanita MC-780 MA) equipped with 8 electrodes and operating at frequencies of 5 kHz, 50 kHz, and 250 kHz. The following parameters were assessed: body fat percentage (FATP) and fat mass in kilograms (FATM); visceral adipose tissue (VAT) on a scale from 0 to 20 (level); muscle mass (percentage and absolute values in kilograms, with regional analysis of upper and lower limbs); fat–free mass (FFM) including total body water correction; body mass index (BMI); and phase angle (PhA). Additionally, total body water (TBW) was evaluated and subdivided into extracellular (ECW) and intracellular water (ICW), from these ECW/TBW ratios were calculated. The literature considers an ECW/TBW ratio of 0.38 to be physiological in healthy individuals [6]. Several sarcopenia-related indices were also calculated: appendicular skeletal muscle mass (ASM) in kilograms, ASM adjusted for height squared (ASM/height^2^), BMI (ASM/BMI), and body weight (ASM/BW). Some indicators were corrected for water distribution using the following formula: ASM – (ECW – 0.38 × (ICW / 0.62)) [6].

Body cell mass (BCM) was computed as FFM minus ECW and bone mass. Participants were classified into six BMI categories: Underweight (< 18.5 kg/m^2^); Normal weight (18.5–24.9 kg/m^2^); Overweight (25–29.9 kg/m^2^. Obesity class I (30–34.9 kg/m^2^); Obesity class II (35–39.9 kg/m^2^); Obesity class III (≥40 kg/m^2^). PhA was considered normal if it ranged between 6.19° and 8.83° in men and 5.98° and 8.04° in women [4].

Statistical analysis was performed using RStudio software with the packages dplyr, ggplot2, and hrbrthemes. To assess the relationship between PhA and body composition variables, the following statistical tests were used: Student’s paired t-test and F-test for continuous variables, and the chi-square goodness-of-fit test for categorical variables. Pearson correlation coefficients were calculated to evaluate the strength of associations between variables. Statistical significance and confidence intervals were determined at a significance level of α = 0.05.

## Results

**Fig. 1:**
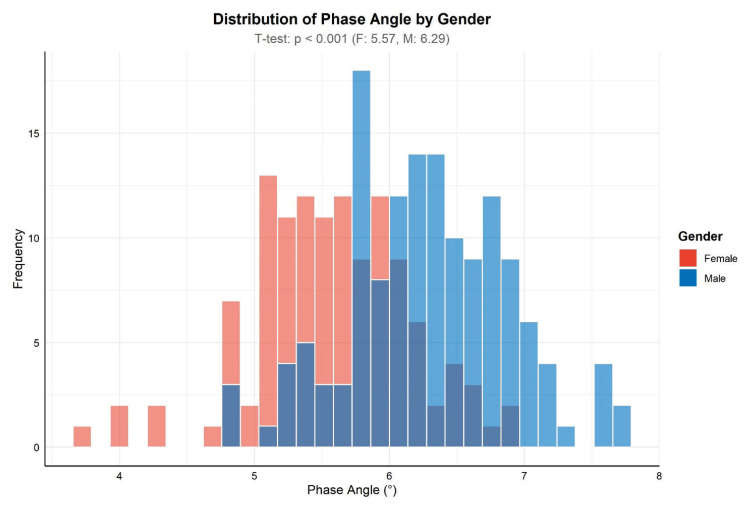
Distribution of phase angle values by sex

The study cohort included 265 patients (122 women and 143 men) with a mean age of 42 years. The most represented group was overweight patients (33 %), with a predominance of males (71 %; p < 0.05). The distribution according to obesity class was as follows: class I obesity - 21 %, class II - 11 %, and class III - 6 %. A statistically significant difference between sexes was found only in class III obesity (4 men vs. 11 women). Normal BMI was observed in 27 % of patients, and only 2 % were underweight. The mean PhA was 5.96° (range 3.79°–7.79°), with men showing higher values (mean 6.29°, range 4.85°–7.79°) than women (mean 5.57°, range 3.79°–6.88°) (Fig. 1).

Men had a higher total body water (TBW) content than women; however, women presented with higher ECW/TBW ratios and lower ICW values. No values indicative of sarcopenia were observed in either sex (criteria: women - ASM < 15 kg, ASM/height < 5.5 kg/m^2^, ASM/BMI < 0.512, ASM/BW < 19.43 %, FFM < 34.7 kg; men - ASM < 20 kg, ASM/height < 7 kg/m^2^, ASM/BW < 0.789, ASM/TH < 25.72 %, FFM < 47.9 kg) [12,13]. Although women had a higher percentage of body fat than men, their values remained within the recommended limits (< 34 % for age), whereas the mean fat percentage in men was 25 %, exceeding the optimal threshold (< 22 % for age). Visceral fat values were within the normal range (≤10) in the whole cohort [14]; however, men had significantly higher levels than women. Patient characteristics are summarized in (Table 1).

**Fig. 2:**
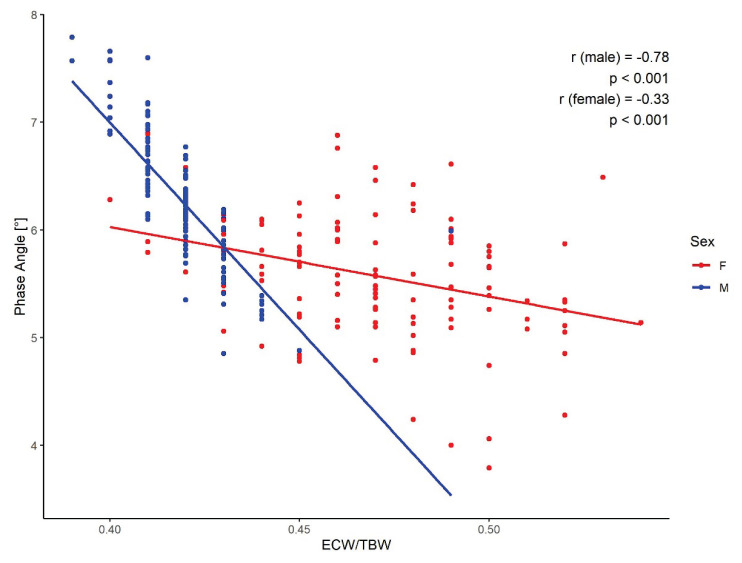
Relationship between PhA and ECW/TBW

Analysis of the relationship between PhA and BIA-derived parameters revealed sex-specific differences. In men, a strong inverse correlation was found between ECW/TBW and PhA (r = −0.78, p < 0.001), while in women, the correlation was weaker (r = −0.33, p < 0.001) (Fig. 2).

**Fig. 3:**
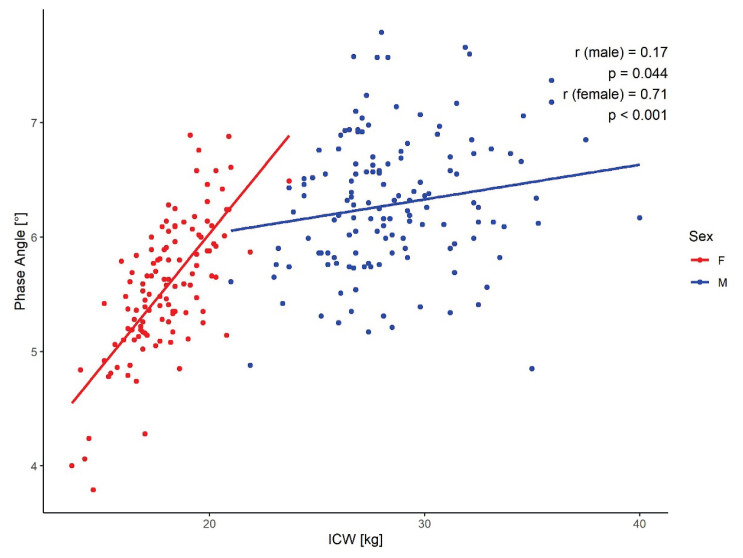
Relationship between PhA and ICW

Conversely, in women a strong positive correlation was observed between ICW and PhA (r = 0.71, p < 0.001), whereas in men this correlation was much weaker (r = 0.17, p < 0.05) (Fig. 3).

Given that elevated ECW may lead to overestimation of muscle mass [6], sarcopenia-related parameters were corrected for ECW. Comparing the correlations of PhA with muscle mass before and after ECW correction showed stronger associations with corrected values, particularly between PhA and ASM/height corrected for ECW (r = 0.61, p < 0.001). The correlation was significantly stronger in women (r = 0.57, p < 0.001) compared to men (r = 0.25, p < 0.003). Overall, more statistically significant correlations were observed in women than in men, suggesting that female patients were the primary contributors to the overall findings (Table 2).

**Fig. 4:**
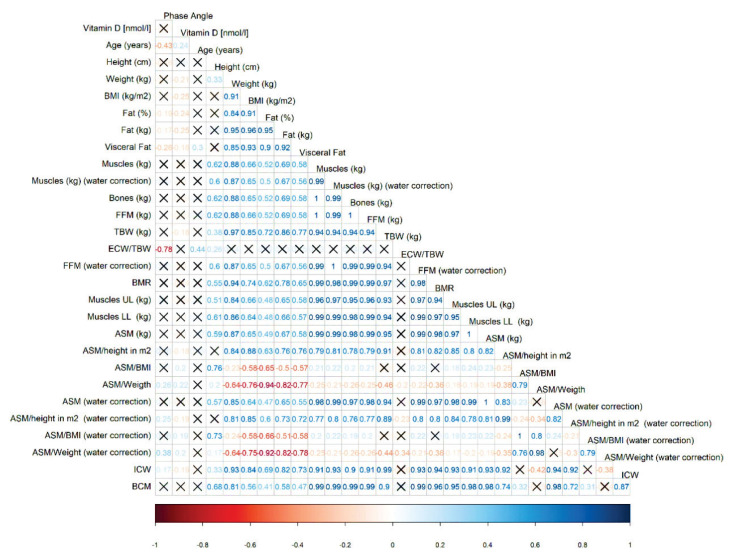
Relationship between all measured and calculated parameters in men

**Fig. 5:**
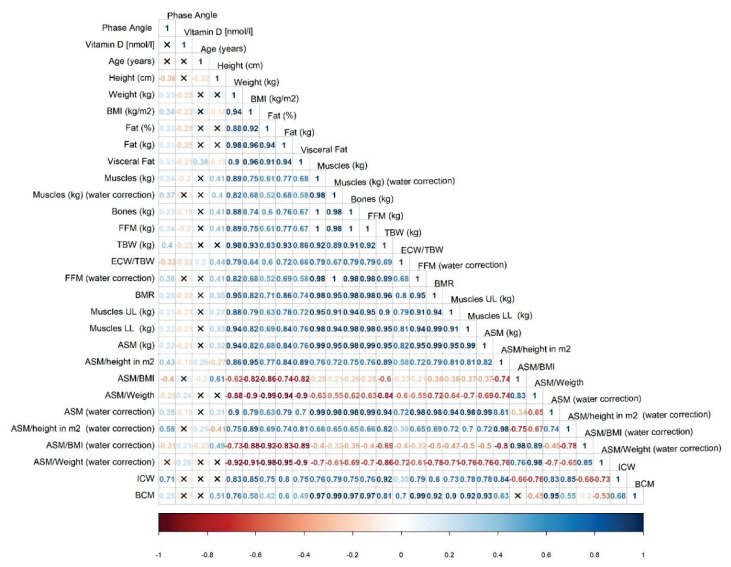
Relationship between all measured and calculated parameters in women

To better visualize the relationships between measured and derived parameters, correlation plots were created separately for men (Fig. 4) and women (Fig. 5). In women, ICW was positively associated with BMI, BW, muscle mass but also with fat mass. Negative correlations were found between ICW and the index ASM/BMI and ASM/BW, indicating that lower values of these indexes were associated with lower ICW. Neither muscle nor fat mass showed a dominant influence on PhA, suggesting that their effects may be balanced. Women had lower ICW and muscle mass, and higher ECW/TBW, indicating that muscle mass (particularly after ECW correction) could be a key determinant of PhA in women.

In men, ECW/TBW appeared to be the main determinant of PhA. This parameter was positively influenced by age (older age = higher ECW/TBW) and negatively correlated with ASM/height after ECW correction (higher ECW/TBW = lower ASM/height). Unlike in women, PhA in men was more strongly related to fat mass - especially visceral fat - than to muscle mass, which showed no significant correlation. Since ECW/TBW was not correlated with fat mass in men, a regression model (R^2^ = 0.63) was used to assess whether ECW/TBW or visceral fat had a greater impact on PhA. Both factors were statistically significant, but ECW/TBW (β = − 37.403; p < 0.001) had a greater effect than visceral fat (β = − 0.018; p < 0.004).

## Discussion

Body composition analysis can be performed using methods such as CT, MRI, or DXA; however, these techniques are expensive and often not readily accessible to the general population. For this reason, bioelectrical impedance analysis (BIA) is frequently used in routine clinical practice, as it is more affordable and widely implemented in outpatient settings. BIA technology has undergone significant development, from the original single-frequency measurements (50 kHz) introduced in the 1980s to the current multifrequency devices, which allow for more accurate differentiation of body compartments, including the division of total body water (TBW) into extracellular (ECW) and intracellular (ICW) components. Advanced BIA devices also measure PhA, which is considered an indicator of overall physical condition and cellular health. Despite inconsistent conclusions in the literature about which BIA parameters most significantly impact PhA, intracellular water (ICW), extracellular water (ECW), fat–free mass (FFM), and body fat are commonly reported as influential factors [11].

Most studies have focused on the use of PhA to assess nutritional status and prognosis in hospitalized patients or individuals with chronic conditions, including malignancies, sarcopenia, renal insufficiency, or chronic inflammatory diseases. In contrast, research in healthy populations is limited, methodologically inconsistent, and often constrained by small sample sizes. Our study stands out in this context: it analyzes a large cohort of 265 healthy adults and focuses on the relationship between PhA and detailed body composition parameters, with a particular emphasis on sex-related differences. The device used (Tanita MC-780 MA) provides multifrequency segmental analysis using eight electrodes and three frequencies (5, 50, and 250 kHz), enhancing the accuracy and clinical relevance of the data. Measurements were taken under standardized conditions in an outpatient setting, reflecting real-world clinical practice. These aspects make our study a valuable contribution to the understanding of factors influencing PhA in a healthy adult population.

### Phase angle and sex

In the studied cohort of healthy adults with a mean age of 42 years, we recorded average PhA values of 6.29° for men and 5.57° for women. Sex-based differences in PhA are consistently reported in the literature, with differences ranging from 0.32° to 0.63° [9]. The reference values for the 50th percentile for a comparable age group is 7.2°–7.3° for men and 6.2°–6.3° for women, while the 25th percentile is set at 6.5° for men and 5.6° for women [15]. When comparing our results with these normative data, it is evident that the average PhA values in our population are slightly lower, especially for men. Barbosa-Silva *et al*. report an average PhA of 6.93° ± 1.15° for healthy volunteers; 7.48° ± 1.10° for men and 6.53° ± 1.01° for women [16]. Campa *et al*., in an athletic population, report average values of 7.4°–7.7° for men and 6.5°–6.9° for women, noting that values vary by sport type and further stratifying PhA values by percentiles [17]. Younger populations tend to show higher PhA values, as demonstrated by the study by Ballarin *et al*., which reports average values of 7.1° for men and 5.9° for women in university students (mean age 24 years), emphasizing the potential of PhA as a predictor of physical fitness [10]. Available evidence suggests that the menstrual cycle has only a minor and inconsistent effect on bioelectrical impedance parameters, including phase angle (PhA). Short-term fluid fluctuations, particularly in physically active women, may slightly affect resistance (R) and reactance (Xc), whereas PhA remains stable, indicating that hormonal cycles do not systematically influence intra- and extracellular water distribution [18].

### Phase angle and age

Campa *et al*. investigated age-related trends in PhA and observed an increase in PhA values in the male population up to approximately 33 years of age, followed by a gradual decline. This decline became statistically significant particularly after the age of 51. In females, a decrease in PhA was noted starting at 37 years of age, with values stabilizing around the age of 58 [15]. However, it is important to emphasize that physical activity – especially strength (resistance) training – may contribute to increasing PhA values and delay or mitigate the physiological decline associated with inactivity and aging. In our cohort, the mean age was 42 ± 12.4 years, representing a relatively narrow age range, thus limiting the opportunity to detect more significant age-related changes in PhA across the entire cohort. Upon closer analysis of the male subgroup, a statistically significant negative correlation between age and PhA values was noted. This relationship can likely be explained by age-related changes in body composition, particularly a decrease in muscle mass along with a relative increase in fat mass, with emphasis on visceral fat. Visceral fat has long been associated with an unfavorable metabolic profile, increased inflammatory activity, and negative effects on cell membrane integrity. For these reasons, it may represent a significant factor contributing to the decrease in PhA values as an indicator of cellular health and the functional status of body tissues.

### Phase angle and body fluid distribution

A statistically significant negative correlation was observed between PhA and the extracellular water-to-total body water ratio (ECW/TBW) (r = − 0.64), with an average ECW/TBW of 0.44 in our cohort. This relationship aligns with findings from Martins *et al*. (r = − 0.64) and Mullie *et al*. (r = − 0.79) [19,20]. Del Giorno *et al*. [21] also reported decreasing ECW/TBW values across increasing PhA quartiles in a mixed-diagnosis cohort. However, these studies rarely stratified by sex. In our analysis, men demonstrated a stronger negative correlation (r = − 0.78), suggesting a greater contribution to the overall association. Conversely, PhA positively correlated with intracellular water (ICW) (r = 0.59), most notably in women (r = 0.71), a trend similarly reported in the literature (Martins *et al*. [19]: r = 0.73; Pérez *et al*. [22]: r = 0.37 in palliative patients). These findings support the concept that PhA reflects not only total fluid volume but also its compartmental distribution, with higher ICW and lower ECW associated with better cellular function and higher PhA values. Body composition significantly influences fluid distribution. Fat tissue exhibits a markedly higher ECW/ICW ratio than fat–free mass (up to 3.7 vs. ~0.8 in FFM), potentially contributing to PhA variability. Additionally, elevated ECW can result from fluid retention, inflammation, or metabolic disturbances such as obesity, insulin resistance, or malnutrition [23,24]. On the other hand, physiological stimuli, particularly endurance or resistance training, can increase ICW and improve cellular integrity and metabolic activity [2].

### Phase angle and muscle mass

Given its high-water content (70–85 %) and dense cellular structure, muscle mass is a major determinant of bioelectrical impedance. It has low resistance and high reactance, both critical components in PhA calculation. Therefore, the quantity and integrity of muscle tissue significantly affect PhA values. In our cohort, PhA correlated positively with fat–free mass (FFM) (r = 0.46), with the correlation improving after correction for ECW (r = 0.56). This agrees with earlier findings, such as those by Skrzypek *et al*. (r = 0.73) [25], and the systematic review by Martins *et al*., where 32 of 59 studies confirmed a positive relationship between muscle mass and PhA [11]. These data support the use of PhA as a proxy marker for muscle quality and integrity, with relevance for diagnosing sarcopenia and monitoring nutritional status.

### Phase angle and fat mass

In our study, fat mass was not identified as a significant determinant of PhA values. Although we found a weak negative correlation between total fat mass and PhA (r = − 0.22), this relationship cannot be considered clinically significant. Consistent with the results of Gonzalez *et al*. [26], we observed a negative correlation in men (r = − 0.19) and a positive correlation in women (r = 0.22) when stratifying by sex. A more pronounced inverse relationship between fat mass and PhA was described by Barrea *et al*. in patients with Prader-Willi syndrome, where the correlation coefficient reached r = − 0.60 [27]. According to a meta-analysis involving 59 studies, 31 studies investigated the relationship between body fat and PhA, 11 detected an inverse relationship, 9 a positive relationship, and 11 found no significant relationship [11]. The inconsistency of results may be partially explained by methodological differences in assessing fat mass, whether through anthropometric measurements or bioelectrical impedance analysis, as well as varying fat distribution in the body, particularly subcutaneous versus visceral fat. Unlike subcutaneous adipose tissue, increased accumulation of visceral fat is associated with greater macrophage infiltration and the release of pro-inflammatory cytokines (TNF-α, IL-6) and adipokines (e.g., leptin). This process promotes low-grade systemic inflammation, impairs cell membrane integrity, and increases the extracellular water fraction (ECW/TBW), which is reflected by a reduction in phase angle (PhA) [2,28].

Visceral fat tissue (VAT) has repeatedly been shown in studies as a factor negatively affecting PhA values. Siddiqui *et al*. describe an indirect relationship between the percentage of VAT and PhA in young healthy athletes [29]. Ferraz *et al*. [28] reports an inverse relationship between VAT and PhA in cardiology patients, with a predictive cutoff of PhA ≤ 5.59° for increased VAT. When stratified by sex, this threshold was associated with a higher risk of VAT in women, while in men, a lower PhA cutoff of ≤ 4.82° was established. These authors report a correlation of r = − 0.32 between PhA and VAT [27,28]. In contrast, in our study, we identified a weaker correlation between these parameters (r = 0.16), and upon stratification by sex, we observed positive relationship in women (r = 0.21; average VAT 6.63) and negative relationship in men (r = − 0.26; average VAT 10.88), which may indicate a pro-inflammatory effect of VAT on PhA. Krivušová et al. reported that the association between VAT and age is more pronounced in women than in men [30]. This finding is consistent with our results, as women in our cohort (mean age 40 years) did not yet exhibit increased VAT compared with men of the same BMI. It can be assumed that with the onset of menopause and the decline in estrogen levels, a substantial shift in fat distribution occurs in women towards a higher proportion of VAT, which may have important clinical implications. These differing trends may reflect sex-specific fat distribution and different physiological limits, with our results also confirming that increased VAT may be associated with a decrease in PhA, as noted by Ferraz *et al*. [28].

### Phase angle and Body mass index, Body cell mass

The relationship between BMI and PhA has been a subject of discussion in the scientific literature, with no clear consensus. According to Barrea *et al*., an increase in BMI results in a linear decrease in PhA, with each unit increase in BMI being associated with a reduction of approximately 0.54° in PhA across different body weight categories [31]. In contrast, Fu *et al*. found a positive relationship between BMI and PhA in overweight and obese individuals, with higher BMI associated with higher PhA values [32]. These differing results likely reflect the heterogeneity of the studied populations and variations in the proportion of active body mass. However, in our analyzed cohort, BMI did not prove to be a significant correlational parameter in relation to PhA, which may be due to the relative homogeneity of the group in terms of BMI.

On the other hand, Body Cell Mass (BCM), which excludes extracellular water and represents the active metabolic component of the body, showed a positive correlation with PhA (r = 0.49), which is consistent with the results of most published studies. For example, Martins *et al*. report a stronger correlation (r = 0.732), confirming the close relationship between cellular integrity and the amount of functionally active tissue [19]. Del Giorno *et al*. [21] describe that patients with PhA values < 4° have significantly reduced BCM, similar to De Franca *et al*. [33], who observed a similar trend at PhA values < 6.3°.

A strong point of our observational study is its focus on a healthy outpatient population, allowing for the definition of reference characteristics for PhA in the context of everyday practice and physiological ranges. According to available meta-analysis, most published studies have primarily focused on patients with chronic diseases (45.76 %), while only 22.03 % pertain to healthy populations [11]. Therefore, our work significantly contributes to filling the data gap in this relatively neglected area. Another strength is the range of body composition parameters analyzed using BIA, including the evaluation of the extracellular to intracellular water ratio, a well-known determinant of PhA. Unlike many previous studies, we also conducted a detailed gender-specific analysis, which is necessary due to the established physiological differences in body composition and PhA values.

While this study provides valuable insights into the role of PhA in a healthy population, it is limited by the absence of detailed information on lifestyle factors, such as physical activity levels and nutritional habits. These factors are crucial in understanding the full scope of body composition and cellular health. Future research that incorporates these variables, as well as prospective studies to monitor changes in PhA over time, will enhance our understanding of PhA as a biomarker for overall health, aging, and disease.

## Conclusion

This study demonstrates that phase angle (PhA), as measured by bioelectrical impedance analysis (BIA), closely reflects body composition and fluid distribution in healthy adults, with distinct sex–specific determinants. The strongest determinant of PhA in men was the extracellular water-to-total body water ratio (ECW/TBW), while in women, intracellular water (ICW) and muscle mass, especially after ECW correction, were more influential. These findings reinforce the value of PhA as a sensitive, non-invasive marker of cellular health, functional tissue integrity, and metabolic condition. By focusing exclusively on a healthy outpatient population, this research fills an important gap in the existing literature, which has largely concentrated on clinical or hospitalized cohorts. Our results also highlight the need to consider physiological differences between sexes when interpreting PhA values, particularly in preventive or performance-based settings. Given its responsiveness to fluid shifts and tissue composition, PhA holds strong potential as a practical biomarker for assessing nutritional status, guiding physical training, or monitoring the early signs of metabolic imbalance. Future studies should incorporate longitudinal designs and include data on physical activity, dietary intake, and lifestyle factors to further clarify the role of PhA in health assessment and personalized medicine.

## Figures and Tables

**Table 1:** Patient characteristics

	All n = 265 (Mean ± SD)	Males n = 143 (Mean ± SD)	Females n = 122 (Mean ± SD)	* p < 0.05
*Age (years)*	42.23 ± 12.39	43.62 ± 12.52	40.59 ± 12.08	*
*Height (cm)*	174.05 ± 10.83	181.31 ± 7.58	165.55 ± 7.30	*
*Body weight (BW) (kg)*	87.91 ± 20.9	95.03 ± 18.55	79.57 ± 20.47	*
*Body mass index (BMI) (kg/m* *^2^* *)*	28.99 ± 6.46	28.90 ± 5.44	29.10 ± 7.51	NS
*Phase angle (PhA) (°)*	5.96 ± 0.69	6.29 ± 0.60	5.57 ± 0.57	*
*Fat (%)*	28.99 ± 9.35	25,00 ± 7.16	33.66 ± 9.47	*
*Fat (kg)*	26.53 ± 13.30	24.87 ± 11.85	28.47 ± 14.63	*
*Visceral fat (level)*	8.92 ± 5.05	10.88 ± 4.99	6.63 ± 4.08	*
*Fat free mass (FFM) (kg)*	61.38 ± 12.22	70.15 ± 8.25	51.10 ± 6.96	*
*FFM (correction for water)*	57.38 ± 12.50	67.10 ± 7.87	46.00 ± 5.10	*
*Muscle (kg)*	58.31 ± 11.65	66.68 ± 7.87	48.50 ± 6.61	*
*Muscle (kg) (correction for water)*	54.32 ± 11.94	63.63 ± 7.49	43.4 ± 4.76	*
*Muscles of the upper limb (kg)*	6.43 ± 2.09	8.01 ± 1.46	4.58 ± 0.79	*
*Muscles of the lower limb (kg)*	19.44 ± 4.86	22.88 ± 3.74	15.41 ± 2.20	*
*Total body water (TBW) (kg)*	42.30 ± 9.10	49.21 ± 5.73	34.21 ± 4.45	*
*Extracellular water to total body water ratio (ECW/TBW) (kg)*	0.44 ± 0.04	0.42 ± 0.01	0.47 ± 0.05	*
*Intracellular water (ICW) (kg)*	23.75±5.96	28.62±3.97	18.04±1.77	*
*Appendicular skeletal muscle mass (ASM) (kg)*	25.97 ± 6.74	31.17 ± 4.46	19.99 ± 2.94	*
*Appendicular skeletal muscle mass adjusted for body weight (ASM/BW)*	0.30 ± 0.05	0.33 ± 0.03	0.26 ± 0.03	*
*Appendicular skeletal muscle mass adjusted for height squared (ASM/Height) (kg/m* *^2^* *)*	8.46 ± 1.52	9.44 ± 1.11	7.31 ± 1.06	*
*Appendicular skeletal muscle mass adjusted for body mass index (ASM/BMI)*	0.91 ± 0.23	1.09 ± 0.15	0.71 ± 0.11	*
*ASM (correction for water)*	24.23 ± 6.82	29.68 ± 4.26	17.88 ± 2.11	*
*ASM/Height in m* *^2^* * (correction for water)*	7.87 ± 1.56	9.01 ± 1.08	6.54 ± 0.81	*
*ASM/BMI (correction for water)*	0.85 ± 0.24	1.04 ± 0.14	0.64 ± 0.11	*
*ASM/BW (correction for water)*	0.28 ± 0.05	0.32 ± 0.03	0.23 ± 0.04	*
*Body cell mass (BCM) (kg)*	39.80 ± 8.41	46.09 ±5.60	32.50 ± 4.13	*

**Table 2:** Correlation between PhA and BIA parameters

	Correlation without gender difference n = 264	* p < 0.05	Males n = 142	* p < 0.05	Females n = 122	* p < 0.05
*Age (years)*	−0.18	*	−0.43	*	−0.12	
*Height (cm)*	0.23	*	−0.16		−0.36	*
*Body Weight (BW) (kg)*	0.24	*	−0.11		0.23	*
*Body mass index (BMI) (kg/m* *^2^* *)*	0.13	*	−0.05		0.34	*
*Fat (%)*	−0.22	*	−0.19	*	0.22	*
*Fat (kg)*	−0.05		−0.17	*	0.21	*
*Visceral fat (level)*	0.16	*	−0.26	*	0.21	*
*Fat–free mass (FFM) (kg)*	0.46	*	0.01		0.24	*
*FFM (correction for water)*	0.52	*	0.09		0.36	*
*Muscle (kg)*	0.46	*	0.01		0.24	*
*Muscle (kg) (correction for water)*	0.53	*	0.10		0.37	*
*Muscles of the upper limb (kg)*	0.50	*	0.10		0.21	*
*Muscles of the lower limb (kg)*	0.46	*	0.00		0.22	*
*Total body water (TBW) (kg)*	0.52	*	0.03		0.40	*
*Extracellular water to total body water ratio (ECW/TBW) (kg)*	−0.64	*	−0.78	*	−0.33	*
*Intracellular water (ICW) (kg)*	0.59	*	0.17	*	0.71	*
*Appendicular skeletal muscle mass (ASM) (kg)*	0.48	*	0.03		0.22	*
*Appendicular skeletal muscle mass adjusted for body weight (ASM/BW)*	0.40	*	0.26	*	−0.24	*
*Appendicular skeletal muscle mass adjusted for height squared (ASM/Height) (kg/m* *^2^* *)*	0.53	*	0.16		0.43	*
*Appendicular skeletal muscle mass adjusted for body mass index (ASM/BMI)*	0.37	*	0.07		−0.39	*
*ASM (correction for water)*	0.52	*	0.11		0.34	*
*ASM/Height in m* *^2^* * (correction for water)*	0.61	*	0.25	*	0.57	*
*ASM/BMI (correction for water)*	0.43	*	0.16		−0.31	*
*ASM/BW (correction for water)*	0.47	*	0.38	*	−0.14	
*Body cell mass (BCM) (kg)*	0.49	*	0.06		0.25	*

## Abbreviations

ASM: Appendicular skeletal muscle mass
ASM/BMI: Appendicular skeletal muscle mass adjusted for body mass index
ASM/BW: Appendicular skeletal muscle mass adjusted for body weight
ASM/height^2^: Appendicular skeletal muscle mass adjusted for height squared
BCM: Body cell mass
BIA: Bioelectrical impedance analysis
BMI: Body mass index
CT: Computed tomography
DXA: Dual-energy X-ray absorptiometry
ECW: Extracellular water
ECW/TBW: Extracellular water to total body water ratio
FATM: Fat mass (in kilograms)
FATP: Fat percentage
FFM: Fat–free mass
FM: Fat mass
ICW: Intracellular water
ICW/ECW: Intracellular to extracellular water ratio
IL-6: Interleukin 6
MRI: Magnetic resonance imaging
PhA: Phase angle
R: Resistance
R^2^: Coefficient of determination (statistical regression parameter)
TBW: Total body water
TNF-α: Tumor necrosis factor alpha
VAT: Visceral adipose tissue
Xc: Reactance
